# Analysis of the Relationship Between *CHRNA*3/5 and *EPHX1* Polymorphisms to Tobacco Intake and Development of Chronic Obstructive Pulmonary Disease (COPD)

**DOI:** 10.3390/biomedicines13112781

**Published:** 2025-11-14

**Authors:** Thiago Prudente Bartholo, Luis Cristóvão Porto, Roberto Pozzan, Adriana Nascimento, Barbara Beatriz Garcia Raskovisch Bartholo, Rogerio Rufino, Cláudia Henrique da Costa

**Affiliations:** 1 Thorax Department, State University of Rio de Janeiro, Rio de Janeiro 20550-013, RJ, Brazil; rpozzan@globo.com (R.P.); barbarabgrm@hotmail.com (B.B.G.R.B.); rrufino.uerj@gmail.com (R.R.); ccosta.uerj@gmail.com (C.H.d.C.); 2 Laboratory of Histocompatibility and Cryopreservation, State University of Rio de Janeiro, Rio de Janeiro 20550-013, RJ, Brazil; luis.cristovaoporto@gmail.com (L.C.P.); drikapn@yahoo.com.br (A.N.)

**Keywords:** COPD, Tobacco intake, *CHRNA* polymorphism, *EPHX1* polymorphism

## Abstract

**Background:** Chronic obstructive pulmonary disease (COPD) is a complex condition influenced by both environmental and genetic factors. Among the genetic determinants, polymorphisms in the *CHRNA3/5* and *EPHX1* genes have been implicated in nicotine dependence and susceptibility to COPD in several populations. However, evidence remains limited in admixed populations such as Brazilians. **Methods:** This cross-sectional study investigated the association between *CHRNA3* (rs1051730, rs8034191) and *EPHX1* (rs2234922) polymorphisms with tobacco nicotine dependence and COPD in a Brazilian cohort. Genotyping was performed using TaqMan^®^ SNP assays, and pulmonary function was assessed via spirometry according to ATS/ERS standards. Associations between genetic variants, tobacco intake, and COPD status were evaluated using χ^2^ and Fisher’s exact tests, with odds ratios (ORs) and 95% confidence intervals (CIs). Post hoc power analyses were conducted to estimate detectable effect sizes. **Results:** A total of 123 active or former smokers were analyzed. The *CHRNA3* variants (rs1051730 and rs8034191) showed a trend toward higher prevalence among individuals with heavy tobacco intake (>40 pack-years), though no significant allelic or genotypic differences were found between COPD and control groups (*p* > 0.05). The *EPHX1* rs2234922 A allele was significantly more frequent in COPD patients, suggesting increased disease risk (*p* < 0.05), while the GG genotype appeared protective. Post hoc power analyses indicated moderate power (≈0.56–0.63) for the observed associations. **Conclusions:** In this Brazilian population, the *CHRNA3/5* polymorphisms may influence nicotine dependence, while *EPHX1* rs2234922 appears to be associated with COPD susceptibility. These findings support a potential genetic contribution to disease risk and tobacco nicotine dependence, warranting further large-scale studies to confirm these associations and explore their therapeutic implications.

## 1. Introduction

Chronic obstructive pulmonary disease (COPD) is a leading cause of morbidity and mortality worldwide and remains one of the most preventable chronic diseases associated with tobacco exposure. It is characterized by progressive and largely irreversible airflow limitation resulting from an abnormal inflammatory response of the lungs to harmful particles or gases, primarily cigarette smoke [1]. The development of COPD in smokers, however, is highly heterogeneous—only a subset of chronic smokers develop clinically significant disease—suggesting that genetic susceptibility plays a major role in determining individual risk [2,3].

Among the biological mechanisms implicated in COPD pathogenesis, oxidative stress plays a central role in amplifying airway inflammation, promoting epithelial apoptosis, and impairing tissue repair [4,5]. The *EPHX1* gene, located on chromosome 1q42.1, encodes microsomal epoxide hydrolase (mEH), an enzyme responsible for detoxifying reactive epoxide intermediates produced during the metabolism of xenobiotics, including those from cigarette smoke [6]. The *EPHX1* rs2234922 (A>G) polymorphism leads to a His139Arg substitution that alters enzyme catalytic efficiency, potentially increasing the burden of oxidative damage in pulmonary tissues [7]. Carriers of the “slow” allele exhibit reduced enzymatic activity and may be more susceptible to smoking-related airway injury and COPD development [8]. Therefore, *EPHX1* polymorphisms represent functional variants that may modulate individual responses to oxidative stress and influence disease progression. A systematic search regarding *EPHX1* gene showed interesting results [9]. While the *EPHX1* rs1051740 polymorfism was associated with high COPD risk among Asians and Caucasians, the *EPHX1* rs2234922 was associated with a protection against COPD in Asians, but not in Caucasians [10]. These studies were carried out in Caucasian and Asian populations, with very little information on genetic polymorphisms in the Brazilian population and none of them addressing their relationship with COPD susceptibility.

In parallel, nicotinic acetylcholine receptor (nAChR) genes located in the *CHRNA3*/*CHRNA5*/*CHRNB4* cluster on chromosome 15q25.1 have been consistently linked to smoking behavior, nicotine dependence, and COPD risk [11,12,13]. The *CHRNA3* and *CHRNA5* polymorphisms are known to influence the affinity and response of nicotinic receptors to nicotine, modulating both the intensity of tobacco use and the reinforcing properties of smoking [14]. Consequently, these genetic variants may contribute indirectly to COPD pathogenesis by increasing lifetime tobacco exposure, independent of direct biological effects on the lung.

Together, these findings support a dual mechanistic model of COPD susceptibility in smokers: (1) direct vulnerability to oxidative stress mediated by *EPHX1* dysfunction, and (2) enhanced tobacco consumption driven by *CHRNA3/5* receptor variants. Understanding the interaction between these genetic pathways and environmental exposure is essential for identifying individuals at higher risk and developing targeted prevention strategies.

Based on this rationale, the present study was designed to test the hypothesis that the *EPHX1* polymorphism increases the risk of COPD development in individuals who smoke, while *CHRNA3/5* polymorphisms contribute to increased tobacco intake, thereby acting synergistically to accelerate the onset and severity of smoking-related pulmonary disease [10,11,12,13,14].

## 2. Materials and Methods

### 2.1. Study Design and Setting

This was a cross-sectional, case–control, genetic-association study conducted at a tertiary, university-affiliated hospital between January and December 2023. The objective was to evaluate the association of *EPHX1* and *CHRNA3/5* polymorphisms with the risk of chronic obstructive pulmonary disease (COPD) and tobacco exposure among adult smokers. The study followed the STREGA (STROBE extension for genetic association studies) recommendations to ensure transparent reporting of genetic epidemiology findings [15].

### 2.2. Participants

Eligible participants were adults (≥18 years) who were current or former smokers with a smoking history of at least 10 pack-years. Two study groups were defined according to spirometric criteria established by the Global Initiative for Chronic Obstructive Lung Disease (GOLD) [1]:

1. COPD group—participants with post-bronchodilator ratio between the forced expiratory volume in one second (FEV_1_) and the forced vital capacity (FVC) < 0.70;
2. Control group—smokers with normal spirometry and no history of chronic respiratory disease.

Participants were recruited consecutively from the hospital’s pulmonary function laboratory and smoking cessation outpatient clinic. Exclusion criteria included: pregnancy; recent thoracic or abdominal surgery (<2 months); acute respiratory infection within 4 weeks; significant cardiac disease (e.g., recent myocardial infarction or decompensated heart failure); cognitive impairment; diagnosis of alpha-1-antitrypsin deficiency (low serum level associated with the presence of genetic mutation); and current use of systemic corticosteroids, bronchodilators, or immunosuppressants within 30 days before enrollment.

### 2.3. Clinical and Demographic Data Collection

All participants underwent a structured interview conducted by trained investigators. The standardized questionnaire captured demographic variables (age, sex, ethnicity, smoking history (age at initiation, cigarettes per day, total pack-years, cessation attempts), occupational exposure, and relevant comorbidities.

### 2.4. Pulmonary Function Testing

Spirometry was performed using a calibrated computerized spirometer (Vitatrace^®^, Codax Ltda, Rio de Janeiro, Brazil) following the American Thoracic Society (ATS) technical standards [16]. Participants refrained from smoking for at least one hour before testing. Parameters obtained included forced vital capacity (FVC), forced expiratory volume in one second (FEV_1_), and the FEV_1_/FVC ratio. Results were expressed as both absolute values and percentages of predicted norms based on reference equation described by Knudson [17]. At least three acceptable maneuvers were recorded for each subject, and the best value was used for analysis. All patients underwent bronchodilation test with salbutamol 400 mcg.

### 2.5. Genetic Analysis

Peripheral venous blood (5 mL) was collected from each participant into EDTA tubes. Genomic DNA was extracted using the QIAamp DNA Blood Mini Kit (Qiagen, Hilden, Germany) according to the manufacturer’s instructions. DNA quality and concentration were determined using a NanoDrop™ ND-1000 spectrophotometer (Thermo Fisher Scientific, Waltham, MA, USA).

Genotyping focused on candidate single-nucleotide polymorphisms (SNPs) previously associated with COPD susceptibility or nicotine dependence:

1. *EPHX1* rs2234922 (A>G), a non-synonymous variant leading to a His139Arg substitution that modulates microsomal epoxide hydrolase activity and influences oxidative stress and epithelial injury;
2. *CHRNA3* rs1051730 (G>A) and *CHRNA5* rs8034191 (C>T), implicated in nicotine dependence and smoking intensity.

Genotyping was performed using TaqMan^®^ SNP Genotyping Assays (Applied Biosystems, Foster City, CA, USA) on a QuantStudio™ 5 Real-Time PCR System. Reaction mixtures contained 10 ng of genomic DNA, 5 µL of TaqMan Genotyping Master Mix, and 0.5 µL of the probe assay in a 10 µL total volume. The thermal cycling conditions were as follows: 95 °C for 10 min, followed by 40 cycles of 95 °C for 15 s and 60 °C for 1 min. Allelic discrimination was automatically determined using QuantStudio™ Design and Analysis Software v1.5. Ten percent of samples were randomly reanalyzed for quality control, yielding a concordance rate of 100%.

### 2.6. Statistical Analysis

Statistical analyses were performed using SPSS Statistics 28.0 (IBM Corp., Armonk, NY, USA). Continuous variables were expressed as mean ± standard deviation (SD) or median (interquartile range [IQR]), depending on data distribution. Normality was assessed with the Shapiro–Wilk test. Comparisons between groups were performed using Student’s *t*-test or Mann–Whitney U test for continuous variables, and χ^2^ or Fisher’s exact test for categorical variables. For genotype and allele frequency comparisons (3 × 2 tables), we examined expected cell counts; Fisher’s exact test was used whenever any expected count was <5, otherwise χ^2^ tests were applied. To mitigate sparsity for rare homozygotes, we also prespecified dominant or recessive genetic models where appropriate. For continuous variables (e.g., age, FEV_1_), we used Welch’s *t*-test (no equal-variance assumption) and confirmed homogeneity of variance with Levene’s test.

Genotype distributions were tested for Hardy–Weinberg equilibrium (HWE) using χ^2^ tests. Logistic regression models were used to estimate odds ratios (ORs) and 95% confidence intervals (CIs) for associations between polymorphisms and COPD risk, adjusting for potential confounders (age, sex, BMI, and cumulative tobacco exposure in pack-years). Statistical significance was defined as *p* < 0.05.

Tobacco intake (TI, equivalent to pack-years) was analyzed both as a continuous variable and dichotomized at 40 pack-years. Subjects with TI > 40 pack-years were classified as tobacco-addicted, whereas those with TI ≤ 40 pack-years were considered non-addicted. Associations between genotypes and nicotine dependence status were tested by Fisher’s exact test (2 × 2 tables), and odds ratios (OR) with 95% confidence intervals were reported.

### 2.7. Ethical Considerations

The study was approved by the Institutional Research Ethics Committee (CAAE 62652722.3.0000.5259). Written informed consent was obtained from all participants prior to inclusion. The study adhered to the ethical principles of the Declaration of Helsinki (2013 revision).

## 3. Results

### 3.1. Association of Polymorphisms with Tobacco Intake

A total of 123 smokers were included and subdivided according to tobacco intake (TI) as non-addicted (TI ≤ 40 pack-years, n = 76) and addicted (TI > 40 pack-years, n = 47). Demographic and functional characteristics of both groups are summarized in Table 1. No statistically significant differences in age or sex distribution were observed between the groups (Welch’s *t*-test, *p* > 0.05).

The distribution of alleles and genotypes for the CHRNA3 polymorphisms (rs1051730 and rs8034191) is shown in Table 2. Although the frequency of the risk alleles A (rs1051730) and C (rs8034191) was higher among individuals with heavy tobacco intake (>40 pack-years), the differences did not reach statistical significance (*p* = 0.11 and *p* = 0.10, respectively). Similarly, genotypic analyses revealed a trend toward a greater prevalence of the AG/AA and CT/CC genotypes in heavy smokers, consistent with previous findings, but without statistical significance (*p* = 0.081 and *p* = 0.062, respectively).

No significant differences were observed for *EPHX1* rs2234922 (A>G) between addicted and non-addicted groups (*p* > 0.05), suggesting that this variant is not related to the degree of tobacco exposure in this population.

The allelic analysis suggested that carriers of the A allele of *CHRNA3* rs1051730 and the C allele of *CHRNA3* rs8034191 had higher odds of heavy tobacco intake (OR > 1), although the 95% CI overlapped 1, indicating that these associations did not reach statistical significance. This trend supports a possible role of *CHRNA3* variants in nicotine dependence.

**Figure 1:**
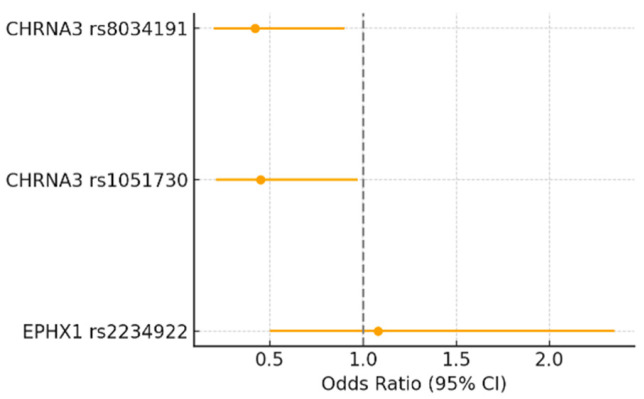
Forest plot showing the odds ratios (95% CI) for the association between *EPHX1* rs2234922, *CHRNA3* rs1051730, and *CHRNA3* rs8034191 polymorphisms and high tobacco intake (TI > 40 pack-years). Odds ratios were calculated under the genetic model showing the strongest association for each polymorphism. The dashed vertical line represents the null value (OR = 1).

Figure 1 illustrates the odds ratios (ORs) and 95% confidence intervals (CIs) for the association between *EPHX1* rs2234922 and *CHRNA3* polymorphisms (rs1051730 and rs8034191) with high tobacco intake (TI > 40 pack-years). Carriers of the A allele of *CHRNA3* rs1051730 and the C allele of *CHRNA3* rs8034191 showed higher odds of belonging to the heavy-smoking group (OR > 1), although the 95% CIs crossed the null line (OR = 1). These trends, while not statistically significant (*p* ≈ 0.06–0.10), are consistent with the hypothesis that *CHRNA3/5* variants may contribute to increased nicotine consumption and nicotine dependence severity. In contrast, *EPHX1* rs2234922 showed no association with tobacco intake (OR ≈ 1), suggesting that this variant does not influence smoking behavior directly.

### 3.2. Association of Polymorphisms with the Development of COPD

The same 123 participants were analyzed according to the presence of airflow obstruction after bronchodilation (FEV_1_/FVC < 0.7). The COPD group (n = 94) consisted of smokers with obstructive spirometry, and the control group (n = 29) included smokers with normal spirometry (FEV_1_/FVC ≥ 0.7).

Demographic and functional data are shown in Table 3. The COPD group contained a higher proportion of male subjects (*p* < 0.05), with no significant difference in mean age. As expected, mean FEV_1_ (% predicted) was significantly lower in the COPD group.

Table 4 shows the allelic distributions, and Table 5 presents the genotypic frequencies and Hardy–Weinberg equilibrium (HWE) results for the polymorphisms studied.

For *EPHX1* rs2234922 (A>G), a significant difference in allele frequency was observed between groups (A allele more frequent among COPD; *p* = 0.010). The genotype distribution also differed significantly (AA, AG, GG; χ^2^ = 6.38, *p* = 0.041), with the AA genotype more prevalent among COPD patients.

For *CHRNA3* rs1051730 (G>A) and *CHRNA3* rs8034191 (C>T), no statistically significant differences were observed between COPD and control groups in either allelic or genotypic comparisons (*p* > 0.05).

Post hoc power analysis (two-proportion approximation, α = 0.05) indicated moderate power to detect the observed differences for *CHRNA3* rs8034191 vs. tobacco intake (power ≈ 0.63) and *EPHX1* rs2234922 vs. COPD (power ≈ 0.56), whereas power was limited for smaller effects. With the current sample (TI ≤ 40, n = 76; TI > 40, n = 47; Controls, n = 29; COPD, n = 94), the minimal detectable absolute differences to achieve 80% power were ~25 percentage points for *CHRNA3*–nicotine dependence contrasts and ~11 percentage points for *EPHX1*–COPD.

## 4. Discussion

In the present study, we observed that the *CHRNA*3/5 polymorphisms (rs1051730 and rs8034191) were associated with a trend toward higher tobacco intake, as individuals carrying the A and C alleles, respectively, were more frequently represented among smokers with cumulative tobacco exposure greater than 40 pack-years. Although these associations did not reach conventional statistical significance in this sample, the direction of effect was consistent with prior evidence linking *CHRNA*3/5 variants to increased nicotine dependence and cigarette consumption.

Therefore, our findings support a potential association between *CHRNA*3/5 variants and higher tobacco intake, suggesting that these polymorphisms may contribute to genetic susceptibility to smoking nicotine dependence. However, given the limited sample size and borderline *p*-values, these results should be interpreted with caution and confirmed in larger cohorts with higher statistical power.

### 4.1. Genetic Susceptibility to Nicotine Dependence

Variants in the *CHRNA*3/5 cluster on chromosome 15q25 have been consistently linked to nicotine dependence and smoking behavior in multiple populations. The *CHRNA*3 rs1051730 and *CHRNA*5 rs16969968 polymorphisms are known to modulate the function of the nicotinic acetylcholine receptor subunits, increasing the reinforcing effects of nicotine exposure and altering smoking intensity [13,18,19]. In our study, smokers carrying the risk alleles A (rs1051730) and C (rs8034191) were more frequent among those with tobacco intake greater than 40 pack-years. This finding aligns with large-scale genome-wide association studies (GWAS) demonstrating that variants within this locus are strongly correlated with smoking quantity and nicotine dependence across different ethnic groups [20,21].

With regard to the correlation between genetic polymorphism and risk of developing COPD, the literature states that some genetic polymorphisms of the *CHRNA*3/5 and EPHX1 gene may be associated with loss of pulmonary function and development of COPD in Caucasian and Asian population, and several studies have associated CHRNA3/5 with an increased risk of pulmonary neoplasia [9,10]. In a sub analysis of the study Evaluation of COPD Longitudinally to Identify Predictive Surrogate Endpoints (ECLIPSE), it was found that polymorphisms of this receptor were significantly associated with the drop in FEV1 and the FEV1/FVC ratio [22]. Among the polymorphisms possibly related to the pathogenesis of COPD, *CHRNA*3/5 rs8034191 and rs1051730 stand out [23].

Although our results reinforce the role of *CHRNA*3/5 polymorphisms in tobacco use, they should be interpreted with caution because genotype frequencies differed modestly between groups, and the observed effect sizes were small to moderate. Furthermore, post hoc power analysis indicated that the study achieved approximately 63% power to detect the observed difference for *CHRNA*3 rs8034191, suggesting that larger samples would be needed to confirm this association with higher confidence. Still, the direction and magnitude of effect observed here are consistent with prior evidence supporting this locus as a determinant of smoking behavior.

### 4.2. EPHX1 Polymorphism and COPD Risk

The *EPHX1* gene encodes microsomal epoxide hydrolase, an enzyme involved in the detoxification of reactive epoxides derived from cigarette smoke and environmental pollutants. Functional polymorphisms in this gene, particularly rs2234922 (His139Arg), have been proposed to modify individual susceptibility to oxidative damage and emphysema [24,25]. In our sample, the *EPHX1* rs2234922 A allele was more frequent among COPD patients, consistent with previous reports suggesting that this variant may reduce enzyme activity and impair detoxification pathways, thereby increasing vulnerability to smoke-induced airway injury.

Nevertheless, the observed association was modest, and the achieved statistical power was 56%, indicating a potential risk of Type II error for smaller effects. Considering our sample of 94 COPD patients and 29 controls, the study could detect an absolute difference of approximately 11 percentage points in carrier frequency with 80% power. Thus, while our results support the biological plausibility of *EPHX1* involvement in COPD susceptibility, replication in larger, multi-center cohorts is necessary to establish definitive evidence.

### 4.3. Studied Population

Although this study was conducted in Rio de Janeiro, the genetic composition of the sample is consistent with that of the broader Brazilian population. Nationwide genomic analyses have demonstrated that individuals from different Brazilian regions share a similar tri-hybrid ancestry pattern, with predominant European and secondary African and Amerindian contributions [26,27]. This genetic homogeneity supports the external validity of our findings and suggests that the associations between *CHRNA*3/5 and *EPHX1* polymorphisms with tobacco intake and COPD risk are likely applicable to other admixed Brazilian populations.

### 4.4. Integration of Genetic and Environmental Factors

COPD is a complex, multifactorial disorder resulting from the interaction between genetic predisposition and environmental exposures such as tobacco smoke, air pollution, and occupational irritants [28]. The coexistence of *CHRNA*3/5 variants influencing smoking behavior and EPHX1 variants affecting detoxification capacity may synergistically enhance risk, leading to higher cumulative exposure and oxidative stress in susceptible individuals. This “dual-pathway” model aligns with recent multi-omics studies showing that nicotine metabolism, inflammatory signalling, and xenobiotic detoxification jointly determine COPD progression [28].

No significant interaction between *CHRNA3/CHRNA*5 and *EPHX1* polymorphisms was detected in relation to either tobacco intake or COPD susceptibility, suggesting that their effects are likely independent and act through distinct biological pathways.

Our findings reinforce this interplay by identifying variants associated with both behavioral and biochemical pathways of disease risk. However, the limited sample size precludes detailed modelling of gene–gene or gene–environment interactions, which would require substantially larger cohorts with standardized exposure quantification.

### 4.5. Power Analysis and Sample Adequacy

The current sample (TI ≤ 40: n = 76; TI > 40: n = 47; Controls: n = 29; COPD: n = 94) provides moderate statistical power for detecting moderate-to-large effect sizes but remains underpowered for subtle genetic effects. Post hoc power analysis demonstrated that the study achieved 63% power for the *CHRNA*3 rs8034191–tobacco intake association and 56% for the *EPHX1* rs2234922–COPD association. To achieve 80% power at α = 0.05, absolute differences of approximately 25 percentage points (for *CHRNA3*–nicotine dependence) and 11 percentage points (for *EPHX1*–COPD) would be required. These estimates suggest that a larger sample—approximately 150 to 200 subjects per group—would be necessary to confirm these findings with robust statistical confidence.

Therefore, while our results are consistent with the direction of previous GWAS and candidate gene studies, the associations observed here should be considered exploratory and hypothesis-generating rather than definitive.

### 4.6. Limitations and Future Perspectives

The main limitations of this study include the relatively small sample size, lack of adjustment for population stratification, and absence of environmental covariates such as occupational exposure or air pollution. The use of candidate gene analysis, while informative, captures only a small fraction of the heritable variance underlying COPD susceptibility and smoking behavior. Future research should integrate polygenic risk scoring, transcriptomic data, and longitudinal follow-up to better characterize causal pathways. In addition, replication in independent populations with diverse ethnic backgrounds will be essential to validate the role of *EPHX1* and *CHRNA*3/5 polymorphisms as predictive biomarkers.

## 5. Conclusions

In conclusion, our study suggests that genetic variants in *CHRNA*3/5 are associated with heavier tobacco use, while *EPHX1* rs2234922 may influence susceptibility to COPD among smokers. The moderate effect sizes and the limited statistical power highlight the need for larger, well-designed studies to confirm these associations and to further explore their potential clinical implications in personalized prevention and smoking-cessation strategies.

## Figures and Tables

**Table 1:** Demographic and functional data according to tobacco intake (TI ≤ 40 vs. >40 pack-years).

Variable	TI ≤ 40 (n = 76)	TI > 40 (n = 47)	*p*-Value
Age (years)	62.4 ± 8.1	64.9 ± 9.3	0.117
Sex (M/F)	48/28	32/15	0.240
FEV_1_ (% predicted)	66.9 ± 23.6	64.9 ± 22.6	0.64

Data are expressed as mean ± standard deviation or frequency. Comparisons were performed using Welch’s *t*-test for continuous variables and χ^2^ or Fisher’s exact test for categorical variables.

**Table 2:** Polymorphisms and tobacco intake (TI ≤ 40 vs. >40 pack-years).

SNP	Risk Allele	TI ≤ 40	TI > 40	*p*-Value
*CHRNA3* rs1051730	A	36/152 (19.2%)	31/94 (24.8%)	0.11
*CHRNA3* rs8034191	C	40/152 (20.8%)	34/94 (26.2%)	0.1
*EPHX1* rs2234922	A	119/152 (78.3%)	72/94 (76.6%)	0.76

Legend: TI: tobacco intake; Odds ratios (OR) and 95% confidence intervals (CI) were calculated. Associations were tested using χ^2^ or Fisher’s exact test according to cell frequencies.

**Table 3:** Demographic and spirometric characteristics by COPD status (Control vs. COPD).

Variable	Control (n = 29)	COPD (n = 94)	*p*-Value
Age (years)	63.1 ± 8.7	66.2 ± 9.1	0.180
Sex (M/F)	14/15	69/25	<0.001
FEV_1_ (% predicted)	94.3 ± 7.8	58.6 ± 13.2	<0.001

Legend: The COPD group was defined by post-bronchodilator FEV_1_/FVC < 0.7. Continuous variables were compared using Welch’s *t*-test, and categorical variables using χ^2^.

**Table 4:** Allelic frequencies of polymorphisms between Control and COPD groups.

Polymorphism	Allele More Frequent in COPD	*p*-Value
*EPHX1* rs2234922 (A>G)	A	0.010
*CHRNA3* rs1051730 (G>A)	—	0.61
*CHRNA3* rs8034191 (C>T)	—	0.85

Legend: The frequency of each allele was compared using Fisher’s exact test. Significant results (*p* < 0.05) are highlighted.

**Table 5:** Genotype frequencies and Hardy–Weinberg equilibrium (HWE) in control group.

Polymorphism	Genotype χ^2^ *p*-Value	HWE χ^2^ (Controls)	HWE *p*-Value
EPHX1 rs2234922 (A>G)	0.041	1.05	0.30
*CHRNA3* rs1051730(G>A)	0.81	0.49	0.48
*CHRNA3* rs8034191 (C>T)	0.85	0.04	0.85

Legend: Genotype distributions were compared between COPD and control groups using χ^2^ tests. HWE was evaluated in controls using the χ^2^ goodness-of-fit test; all SNPs conformed to HWE (*p* > 0.05).

## Data Availability

The original contributions presented in this study are included in the article. Further inquiries can be directed to the corresponding author.
